# Expression and clinical significance of erb-B receptor family in hepatocellular carcinoma

**DOI:** 10.1054/bjoc.2000.1580

**Published:** 2001-05

**Authors:** Y Ito, T Takeda, M Sakon, M Tsujimoto, S Higashiyama, K Noda, E Miyoshi, M Monden, N Matsuura

**Affiliations:** 1Department of Surgery, Osaka Seamen's Insurance Hospital, 1-8-30, Chikko, Minato-ku, Osaka 552-0021, Japan; Department of Pathology,Department of Biochemistry, School of Allied Health Science, Osaka University Faculty of Medicine, 1–7, Yamadaoka, Suita, Osaka 565–0871, Japan; Department of Surgery II, Department of Biochemistry, Osaka University Medical School, 2-2, Yamadaoka, Suita, Osaka 565–0871, Japan; Department of Pathology, Osaka Police Hospital, 10–31, Kitayama-cho, Tennoji-ku, Osaka 543, Japan

**Keywords:** EGF-R, c-erbB-2, c-erbB-3, c-erbB-4, hepatocellular carcinoma, immunohistochemistry

## Abstract

In order to elucidate the clinical significance of the erbB family, epidermal growth factor receptor (EGF-R), c-erbB-2, c-erbB-3 and c-erbB-4 in hepatocellular carcinoma (HCC), we investigated the expression of these proteins by means of immunohistochemistry for HCC as well as adjacent noncancerous lesions. EGF-R was expressed in 68% of the HCC examined and showed correlation with the proliferating activity, stage, intrahepatic metastasis and carcinoma differentiation. c-erbB-2 was expressed in only 21% of the cases and showed no relationships with the clinicopathological parameters. c-erbB-3 protein was observed in 84% of the HCC and 38.1% of the noncancerous lesions. Its expression in HCC was equal to or greater than noncancerous lesions in 90.5% of the cases, and was related to the stage, portal invasion, cell proliferating activity, tumour size, intrahepatic metastasis and carcinoma differentiation. c-erbB-4 protein was expressed in 61.0% of HCC and in as much as 86.1% of the noncancerous lesions. Unlike the expression of c-erbB-3, that of c-erbB-4 in HCC was less than that of the adjacent noncancerous lesions in 51.2% of the cases. No statistical significance could be established between this protein expression in HCC and clinicopathological features. EGF-R and c-erbB-3 affected disease-free survival, but were not recognized as independent prognostic factors by multivariate analysis. The present study suggests that, of the four receptors, EGF-R and c-erbB-3 play important roles in the progression of HCC. © 2001 Cancer Research Campaign www.bjcancer.com


*British Journal of Cancer* (2001) **84**(10), 1377–1383
© 2001 Cancer Research Campaign
doi: 10.1054/ bjoc.2000.1580, available online at http://www.idealibrary.com on IDEAL®

### Expression and clinical significance of erb-B receptor family in hepatocellular carcinoma

**Y Ito[1,2], T Takeda[1], M Sakon[3], M Tsujimoto[4], S Higashiyama[5], K Noda[6], E Miyoshi[6], M Monden[3] and N Matsuura[2]**

[1]Department of Surgery, Osaka Seamen's Insurance Hospital, 1-8-30, Chikko, Minato-ku, Osaka 552-0021, Japan; [2]Department of Pathology, [5]Department of Biochemistry, School of Allied Health Science, Osaka University Faculty of Medicine, 1–7, Yamadaoka, Suita, Osaka 565–0871, Japan; [3]Department of Surgery II, [6]Department of Biochemistry, Osaka University Medical School, 2-2, Yamadaoka, Suita, Osaka 565–0871, Japan; [4]Department of Pathology, Osaka Police Hospital, 10–31, Kitayama-cho, Tennoji-ku, Osaka 543, Japan

**Summary** In order to elucidate the clinical significance of the erbB family, epidermal growth factor receptor (EGF-R), c-erbB-2, c-erbB-3 and c-erbB-4 in hepatocellular carcinoma (HCC), we investigated the expression of these proteins by means of immunohistochemistry for HCC as well as adjacent noncancerous lesions. EGF-R was expressed in 68% of the HCC examined and showed correlation with the proliferating activity, stage, intrahepatic metastasis and carcinoma differentiation. c-erbB-2 was expressed in only 21% of the cases and showed no relationships with the clinicopathological parameters. c-erbB-3 protein was observed in 84% of the HCC and 38.1% of the noncancerous lesions. Its expression in HCC was equal to or greater than noncancerous lesions in 90.5% of the cases, and was related to the stage, portal invasion, cell proliferating activity, tumour size, intrahepatic metastasis and carcinoma differentiation. c-erbB-4 protein was expressed in 61.0% of HCC and in as much as 86.1% of the noncancerous lesions. Unlike the expression of c-erbB-3, that of c-erbB-4 in HCC was less than that of the adjacent noncancerous lesions in 51.2% of the cases. No statistical significance could be established between this protein expression in HCC and clinicopathological features. EGF-R and c-erbB-3 affected disease-free survival, but were not recognized as independent prognostic factors by multivariate analysis. The present study suggests that, of the four receptors, EGF-R and c-erbB-3 play important roles in the progression of HCC. © 2001 Cancer Research Campaign http://www.bjcancer.com

**Keywords**: EGF-R; c-erbB-2; c-erbB-3; c-erbB-4; hepatocellular carcinoma; immunohistochemistry

Hepatocellular carcinoma (HCC) is a common neoplasm especially in East Asia and Africa. Although most HCCs are caused by HBV infection in China (Beasley et al, 1981) and by intake of aflatoxin in Africa (Uwaifo and Bababunmi, 1984), the dominant cause in Japan is HCV (Saito et al, 1990). In spite of enormous efforts to improve clinical treatment, HCC remains a major carcinoma with high mortality. Poor differentiation, larger size, portal invasion and intrahepatic metastasis are known to shorten disease-free survival with this carcinoma.

One of the most prominent parameters in evaluation of the biological aggressiveness of carcinoma is the investigation of cell behaviour. Growth factor receptors with tyrosine kinase activity are known to contribute greatly to the regulation of cell behaviour such as cell growth, proliferation and mortality (Ullrich and Schlessinger, 1990; Cantley et al, 1991). The type I family of growth factor receptors is the most prominent and is recognized as a proto-oncogene family. The family consists of epidermal growth factor receptor (EGF-R), c-erbB-2 and the more recently identified c-erbB-3 and c-erbB-4 (Kraus et al, 1989; Plowman et al, 1993). When specific ligands bind to a receptor of the family, the receptor is activated by phosphorylation of the tyrosine residue in the molecule (Ullrich and Schlessinger, 1990). It then forms a dimer with another receptor of this family, causing activation by transphosphorylation which contributes to a variety of growth signal transductions (Pinkas-Kramarski et al, 1996). These receptors share high sequence identity with each other and are coexpressed in various combinations in neoplasms. Thus far, of the four receptors, the expression of EGF-R and c-erbB-2 has been investigated in various neoplasms, including malignancies of the liver and biliary tract (Brunt and Swanson, 1992; Collier et al, 1992; Nakapoulou et al, 1994; Lee and Pirdas, 1995; Kira et al, 1997; Terada et al, 1998). The other two receptors, c-erbB-3 and c-erbB-4, have only been studied in depth for a few neoplasms (Sanidas et al, 1993; Simpson et al, 1995; Shintani et al, 1995; Haugen et al, 1996; Travis et al, 1996; Bobrow et al, 1997; Bodey et al, 1997; Chow et al, 1997; Ibrahim et al, 1997; Srinivasan et al, 1998, 2000; Suo et al, 1998; Freiss et al, 1999; Haussler et al, 1999; Kapitanovic et al, 2000; Kew et al, 2000), and little is known about their expression in hepatic malignancies. In this study, we investigated the expression of all four components of this family in a large series of HCC to evaluate their clinical significance and to identify the factors reflecting the development of this carcinoma.

*Received 11 May 2000*
*Revised 5 October 2000*
*Accepted 18 October 2000*

*Correspondence to:* N Matsuura

### MATERIALS AND METHODS

### Tissue specimens

10% buffered formalin-fixed paraffin-embedded blocks of HCC were prepared from 100 patients who had undergone surgery for HCC. Informed consent was obtained from each patient. The characteristics of these patients are summarized in Table 1.

**Table 1** Profile of the 100 HCC cases used in this study

| | | | |
|---|---|---|---|
| Age (years) | 62.3 ± 7.5 | | |
| Gender | male | 84 | |
| | female | 16 | |
| Liver cirrhosis | with | 64 | (59) |
| | without | 36 | (25) |
| HCV antibody | with | 66 | (59) |
| | without | 22 | (17) |
| | | (unknown 12) | (unknown 8) |
| HBs antigen | with | 13 | (10) |
| | without | 76 | (66) |
| | | (unknown 11) | (unknown 8) |
| Stage | ≥III | 34 | |
| | <III | 66 | |
| Follow up time (months) | | 22.5 ± 17.2 | |

Figures in parenthesis indicate the profile of 84 patients whom noncancerous lesions were immunohistochemically investigated for erbB receptor family.

### Antibodies

The following antibodies were employed for immunohistochemistry, with the concentrations given in parentheses. Sheep polyclonal antibody against EGF-R (06-129, 1:100) and mouse monoclonal antibody against c-erbB-3 (clone 2F12 1:200) were purchased from UBI (Lake Placid, NY, USA). A mouse monoclonal antibody against c-erbB-2 (clone CB11 1:100) was obtained from Novocastra (London, UK). A rabbit polyclonal antibody against c-erbB-4 (1:500) was established by our coworker and raised against synthetic peptide (35–54 amino acid sequence) of c-erbB-4. We also employed another polyclonal antibody against c-erbB-4 (sc-283, 1:50), which recognizes the same epitope and found that the immunostaining results for these two antibodies were fundamentally identical to each other for the a limited number of cases examined for all diagnostic groups. Human placenta and skeletal muscle were adopted as positive controls for antibodies against c-erbB-3 and c-erbB-4 (Srinivasan et al, 1998). A monoclonal antibody against Ki-67 (clone MIB-1, 1:50) was obtained from Ylem (Rome, Italy).

### Immunohistochemistry

Immunohistochemical study was performed using the avidin-biotin-complex (ABC) method. Briefly, 4-μm slices of tissue section were deparaffinized and endogenous peroxidase activity was blocked with 0.3% hydrogen peroxide and 0.1% sodium azide in distilled water for 15 min. Sections were then incubated with 0.03 mol $L^{-1}$ citrate buffer (pH 6.0) and heated to 121˚C for 20 min in a pressure cooker except for samples to study the immunohistochemistry of c-erbB-4. The sections were next rinsed in phosphate-buffered saline pH 7.2 (PBS), then 10% bovine serum (Wako, Osaka, Japan) was applied for 10 min to block the nonspecific reaction. The sections were incubated with the primary antibody for 60 min at room temperature. After rinsing in PBS, they were treated with biotinylated anti-sheep IgG (Vector, Bulingame, CA, USA) at the concentration of 1:200 for EGF-R, or biotinylated anti-mouse IgG (Amersham, London, UK) at the concentration of 1:200 for c-erbB-2, c-erbB-3 and Ki-67, or biotinylated anti-rabbit IgG (Dako, Copenhagen, Denmark) at the concentration of 1:300 for c-erbB-4 for 15 min. After rinsing again in PBS, the sections were allowed to react with the avidin-biotin peroxidase complex (Dako, Copenhagen, Denmark) at the concentration of 1:300 for 15 min. The peroxidase reaction was visualized by incubating the sections with 0.02% 3,3′-diaminobenzidine tetrahydrochloride in 0.05 M Tris buffer (pH 7.6) with 0.01% hydrogen peroxide. The sections were counterstained with haematoxylin. Sections for the negative control were prepared using normal mouse serum instead of primary antibody.

### Immunohistochemical evaluation

For immunohistochemical evaluation of EGF-R, c-erbB-2, c-erbB-3 and c-erbB-4, we regarded cells positive for these proteins when the immunoreactivity was clearly observed in them. We classified the cases as (++) when more than 50% of the carcinoma cells were positive for these proteins, (+) when 10 to 50% of the cells were positive and (−) when less than 10% of the cells were positive for these proteins. We counted positive cells for Ki-67 by monitoring at least 500 HCC cells and calculated the Ki-67 labelling index (LI). Ki-67 is widely accepted as a prominent marker for cellular proliferation because it is expressed in all cells except for those in the G0 phase (Sasaki et al, 1987). We previously demonstrated that Ki-67 LI showed a prognostic significance for disease-free survival (DFS) of the patients, when the cut-off value was set at 20% (Ito et al, 1999). Similar results were obtained for the series of the present study and were subjected to multivariate analyses of patient survival.

### Survival data

Disease-free survival (DFS) data were analysed for the 85 patients who had undergone curative surgery and could be followed after surgery. They were followed for 5 to 80 months (mean 22.5 months). Postoperative (DFS) curves were constructed by the Kaplan-Meier method.

### Statistical analyses

Values were expressed as mean ± S.D. The chi-squared test and Kruskal-Wallis test followed by Dunn's test of multiple comparisons were employed for analyses of the relationship between the expression of the proteins and various clinicopathological parameters. Univariate DFS data were analysed by the log-rank test. For multivariate analyses, we used the Cox proportional hazard model. A *P* value less than 0.05 was considered to be statistically significant.

### RESULTS

We performed immunostaining for EGF-R, c-erbB-2, c-erbB-3 and c-erbB-4 for 100 HCC cases and 84 noncancerous lesions adjacent to carcinoma nests found in cases of chronic hepatitis with or without liver cirrhosis. Profiles of the patients are shown in Table 1. Various pathological classifications including degree of differentiation conformed to The General Rules for the Clinical and Pathological Study of Primary Liver Cancer of the Liver Cancer Study Group of Japan (Liver Cancer Study Group of Japan, 1992). The TNM staging we adopted in this study was subject to that of the Liver Cancer Group of Japan and is identical to the UICC criteria. Portal invasion and intrahepatic metastasis were histologically diagnosed.

### EGF-R protein expression

EGF-R immunoreactivity was observed mainly in the cell membrane and occasionally and faintly in the cytoplasm of HCC

*© 2001 Cancer Research Campaign*

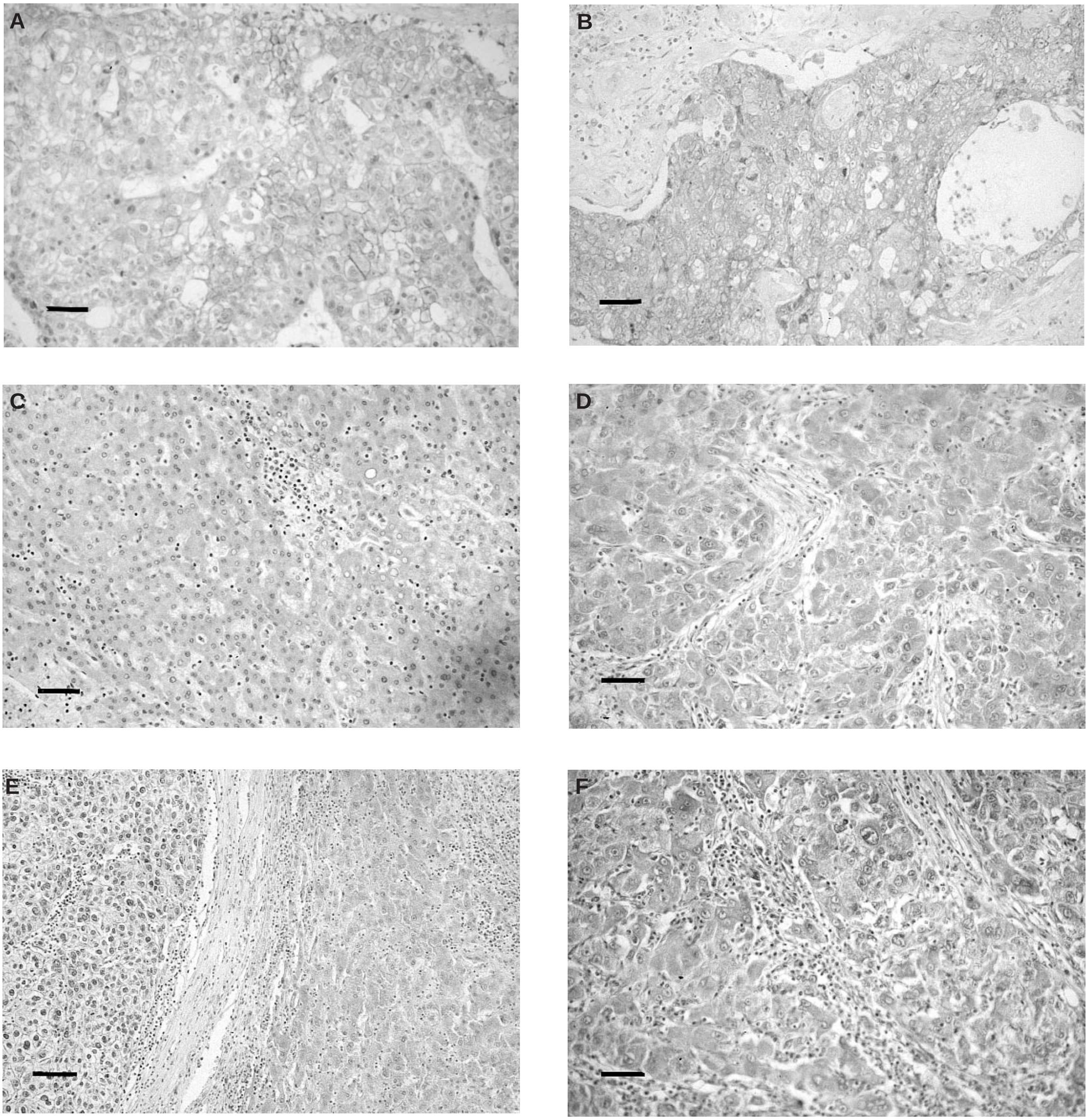

**Figure 1** Immunostaining of the erbB receptor family in HCC and noncancerouslesions. a) EGF-R expression in membranes of HCC cells. b) c-erbB-2 expression in membranes and cytoplasms of HCC cells. c,d) Cytoplasmic c-erbB-3 expression in c) noncancerous lesion and d) HCC. e,f) Cytoplasmic c-erbB-4 expression. e) Diffuse c-erbB-4 expression in noncancerous lesion whereas only equivocal staining in adjacent carcinoma nest which we do not regard 'positive'. f) c-erbB-4 was diffusely expressed in this HCC case. Scale bars: a,b 28 μm; c, d, f 20 μm; e, 40 μm cells and noncancerous hepatocytes. The staining intensity was not definitely different between them. Among the 100 HCC cases, 42 were classified as (++), 26 as (+) and 32 as (−) (Figure 1a). On the other hand, only 13 (15.5%) of the 84 noncancerous lesions were (+) for EGF-R and the remaining cases were (−). In 53 cases, EGF-R was expressed more in HCC than in noncancerous lesions (Table 2). EGF-R was expressed more frequently in HCC cases with high Ki-67 LI ($P < 0.0001$), advanced stage ($P = 0.0435$), intrahepatic metastasis ($P = 0.0101$) and poor differentiation ($P = 0.0190$) (Table 3A). Furthermore, EGF-R expression (++ vs +, −) showed a direct relationship with c-erbB-3 expression (++ vs +, −) ($P = 0.0010$). Furthermore, EGF-R expression (++, +vs−) showed a prognostic significance ($P = 0.0097$) for DFS of the patients in univariate analysis.

**Table 2** Expression of erbB family in HCC

| | Less than noncancerous lesion | Equal to noncancerous lesion | Greater than noncancerous lesion |
|---|---|---|---|
| EGF-R | 0 | 31 | 53 |
| c-erbB-2 | 0 | 68 | 16 |
| c-erbB-3 | 6 | 14 | 64 |
| c-erbB-4 | 43 | 38 | 3 |

### c-erbB-2 protein expression

The immunoreactivity of this protein was observed as membranous staining sometimes with faint cytoplasmic staining. In noncancerous lesions, all cases were negative for this protein. In HCC, no cases were classified as (++), 21 cases as (+) and the remaining 79 were (−) (Figure 1b). As shown in Table 3B, we could not establish any relationship between c-erbB-2 expression and the clinicopathological features as well as the prognosis of the patients.

### c-erbB-3 protein expression

The c-erbB-3 protein was localized mainly in the cytoplasm of the cells both in carcinoma cells and adjacent noncancerous hepatocytes with the similar intensity. Of the 84 cases of noncancerous lesions, 8 cases (9.5%) were (++), 24 cases (28.6%) were (+) and 52 cases (61.9%) were (−) for this protein (Figure 1c). Of the 100 HCC cases, 45, 39 and 16 were classified as (++), (+) and (−), respectively. In 64 cases (76.2%), c-erbB-3 expression in HCC was greater than in noncancerous lesions, whereas 14 cases (16.7%) and only 6 cases (7.1%) expressed this receptor in HCC at a level equal to or less than that in noncancerous lesions, respectively (Table 2). Although c-erbB-3 expression was not related to the stage and portal invasion, it was significantly linked to Ki-67 LI ($P$ = 0.0344), tumour size ($P$ = 0.0202), intrahepatic metastasis ($P$ = 0.0263) and carcinoma differentiation ($P$ = 0.0470) (Table 3C). c-erbB-3 expression (++ vs +, −) showed a prognostic significance for DFS ($P$ = 0.0315) in univariate analysis.

### c-erbB-4 protein expression

The immunoreactivity of c-erbB-4 was localized mainly in the cytoplasm of HCC cells and noncancerous hepatocytes. The immunoreactivity was generally clear in HCC cells and noncancerous hepatocytes, but equivocal staining was sometimes observed in HCC cells which we did not regard as positive. This protein was expressed, unlike the other three proteins, in hepatocytes in noncancerous lesions with very high incidence, that is, 53 cases (63.1%) were (++) and 21 cases (25.0%) were (+) (Figure 1e). On the other hand, among the 100 HCC cases, only 19 were (++), 42 were (+), and the remaining 39 were (−) (Figure 1f). Only 3 (3.6%) of the 84 cases expressed more c-erbB-4 protein in the HCC than in the adjacent noncancerous lesions (Table 2). In 38 cases (45.2%), c-erbB-4 expression level in the HCC was the same as in the adjacent noncancerous lesions. Furthermore, in as many as 43 cases (51.2%), c-erbB-4 expression in HCC was even lower than in the noncancerous lesions. As shown in Table 3D, c-erbB-4 staining in the carcinoma was not linked to any clinicopathological features and prognosis of the patients.

In our series, carcinoma differentiation (poor vs moderate and well) ($P$ = 0.0072), tumour size (≥ 5 cm vs <5 cm) ($P$ = 0.0251), portal invasion ($P$ = 0.0665, borderline significance), intrahepatic metastasis ($P$ = 0.0012) and Ki-67 LI (≥ 20% vs < 20%) ($P$ = 0.0002) also affected the disease-free survival of the patients. We next performed multivariate analysis by means of the Cox hazard proportion model and found Ki-67 LI ($P$ = 0.0405) and intrahepatic metastasis ($P$ = 0.0434) to be independent prognostic factors, whereas EGF-R and c-erbB-3 were not.

### DISCUSSION

Our results showed that EGF-R was very frequently overexpressed in HCC as compared to adjacent noncancerous lesions. Kira et al have demonstrated that the overexpression of EGF-R in HCC was related to carcinoma differentiation (Kira et al, 1997). In the present study, we obtained more informative results probably because of the larger number of cases examined. We found that EGF-R overexpression is also correlated with high proliferating activity, advanced stage, the presence of intrahepatic metastasis and poor disease-free survival. The present study made it clearer that EGF-R strongly reflects the biological aggressiveness of this carcinoma and plays an important role in its progression.

Our results for c-erbB-2 expression in HCC are similar to those of previous studies with a smaller number of cases (Brunt and Swanson, 1992; Collier et al, 1992; Nakopoulou et al, 1994). c-erbB-2 expression was not frequently found and does not contribute to the malignant phenotype in HCC because it was not related to any clinicopathological features including prognosis. Our findings suggest that c-erbB-2 does not play an important role in the progression of HCC, in contrast to several other malignancies (Berchunk et al, 1990; Borg et al, 1990; Kameda et al, 1990; Kern et al, 1990; Dugan et al, 1997; Yang et al, 1997).

This is the first study on the expression of c-erbB-3 and c-erbB-4 in HCC. In the investigation of the protein level for a large number of cases, c-erbB-3 overexpression as compared to noncancerous lesions was observed in 76.2% of the cases. Similar studies have been performed on a few other carcinomas. For example, Sanidas et al demonstrated that the c-erbB-3 protein was always expressed in both gastric carcinoma and the adjacent mucosa, but the expression level was usually higher in the carcinoma (Sanidas et al, 1993). Travis et al observed that breast carcinoma expressed c-erbB-3 more intensely and diffusely than the adjacent normal glands which were usually weakly or moderately positive for this protein (Travis et al, 1996). Haugen et al showed that normal follicles of the thyroid were all negative for c-erbB-3, whereas all types of thyroid carcinoma expressed this protein with very high incidence (Haugen et al, 1996). The results of these studies including ours are similar in that they show c-erbB-3 expression to be more diffuse and/or more intense in the carcinoma nest than in normal or benign lesions.

Our study also showed that c-erbB-3 expression in HCC is significantly related to some important markers of carcinoma progression, which are also predictors of recurrence, such as proliferating activity, tumour size, intrahepatic metastasis and carcinoma differentiation. Furthermore, c-erbB-3 itself, to some extent, affects disease-free survival. Similar results were obtained for breast carcinoma, i.e., c-erbB-3 is related to tumour size and tumour type prognostic group (Travis et al, 1996), and for colorectal carcinoma, i.e., cases without c-erbB-3 expression show a favourable outcome for

 © 2001 Cancer Research Campaign

**Table 3** The relationship between the expression of erbB family and various clinicopathological features of 100 HCC patients

**A. EGF-R expression**

| | | **++ (*n* = 42)** | **+ (*n* = 26)** | **− (*n* = 32)** | |
|---|---|---|---|---|---|
| Ki-67 LI | | 40.4 ± 20.1 | 24.9 ± 17.3 | 20.6 ± 15.1 | * *P* < 0.0001 |
| Stage | ≥III | 19 | 8 | 7 | *P* = 0.0435 |
| | <III | 23 | 18 | 25 | (++vs+, −) |
| Tumour size | ≥5 cm | 22 | 14 | 9 | NS |
| | <5 cm | 20 | 12 | 23 | |
| Portal invasion | with | 19 | 9 | 8 | NS |
| | without | 23 | 17 | 24 | |
| Intrahepatic metastasis | with | 15 | 5 | 3 | *P* = 0.0101 |
| | without | 27 | 21 | 29 | (++ vs +, −) |
| Carcinoma differentiation | poor | 16 | 9 | 1 | *P* = 0.0190 |
| | moderate or well | | 26 | 17 | 31 (++ vs +, −) |
| c-erbB-2 expression | + | 13 | 3 | 5 | |
| | − | 9 | 23 | 27 | NS |
| c-erbB-3 expression | ++ | 27 | 9 | 9 | *P* = 0.0010 |
| | +, − | 15 | 17 | 23 | |
| | ++ | 6 | 6 | 7 | (++ vs+, − ) |
| c-erbB-4 expression | + | 25 | 17 | 20 | NS |
| | − | 11 | 3 | 5 | |

*This analysis was done by Kruskal-Wallis test. *P* = 0.0008 for ++vs+ and <0.0001 for ++vs− by Dunn's test.

| **B. c-erbB-2 expression** | | **+ (*n* = 21)** | **− (*n* = 79)** | |
|---|---|---|---|---|
| Ki-67 LI | | 31.4 ± 21.0 | 29.7 ± 19.5 | NS |
| Stage | ≥III | 8 | 26 | |
| | <III | 13 | 53 | NS |
| Tumour size | ≥5 cm | 11 | 34 | |
| | <5 cm | 10 | 45 | NS |
| Portal invasion | with | 8 | 28 | |
| | without | 13 | 51 | NS |
| Intrahepatic metastasis | with | 3 | 20 | |
| | without | 18 | 59 | NS |
| Carcinoma differentiation | poor | 5 | 21 | |
| | moderate or well | 16 | 58 | NS |
| | ++ | 11 | 34 | |
| c-erbB-3 expression | + | 5 | 34 | |
| | − | 5 | 11 | NS |
| | ++ | 3 | 16 | |
| c-erbB-3 expression | + | 13 | 49 | |
| | − | 5 | 14 | NS |

| **C. c-erbB-3 expression** | **++ (*n* = 45)** | **+ (*n* = 39)** | **− (*n* = 16)** | | |
|---|---|---|---|---|---|
| Ki-67 LI | 31.4 ± 19.2 | 33.1 ± 20.7 | 18.6 ± 13.2 | * *P* = 0.0344 | |
| Stage | ≥III | 19 | 11 | 4 | NS |
| | <III | 26 | 28 | 12 | |
| Tumour size | ≥5 cm | 26 | 16 | 3 | *P* = 0.0202 |
| | <5 cm | 19 | 23 | 13 | (++, +vs−) |
| Portal invasion | with | 20 | 11 | 5 | NS |
| | without | 25 | 28 | 11 | |
| Intrahepatic metastasis | with | 15 | 6 | 2 | *P* = 0.0263 |
| | without | 30 | 33 | 14 | (++, +vs−) |
| Carcinoma differentiation | poor | 15 | 10 | 1 | *P* = 0.0470 |
| | moderate or well | 30 | 29 | 15 | (++, +vs−) |
| c-erbB-4 expression | ++ | 11 | 7 | 1 | |
| | + | 25 | 24 | 13 | NS |
| | − | 9 | 8 | 1 | |

*This analysis was done by Kruskal-Wallis test. *P* = 0.0261 for ++vs- and 0.0261 for +vs - by Dunn's test.

| **D. c-erbB-4 expression** | **++ (*n* = 19)** | **+ (*n* = 62)** | **− (*n* = 19)** | | |
|---|---|---|---|---|---|
| Ki-67 LI | 29.3 ± 14.2 | 29.9 ± 21.2 | 30.9 ± 21.3 | NS | |
| Stage | ≥III | 8 | 21 | 5 | NS |
| | <III | 11 | 41 | 14 | |
| Tumour size | ≥5 cm | 11 | 27 | 7 | NS |
| | <5 cm | 8 | 35 | 12 | |
| Portal invasion | with | 9 | 19 | 8 | NS |
| | without | 10 | 43 | 11 | |
| Intrahepatic metastasis | with | 2 | 18 | 3 | NS |
| | without | 17 | 44 | 16 | |
| Carcinoma differentiation | poor | 6 | 13 | 7 | NS |
| | moderate or well | 13 | 49 | 12 | |

© 2001 Cancer Research Campaign 

| **D. c-erbB-4 expression** | **++ (*n* = 19)** | **+ (*n* = 62)** | **− (*n* = 19)** | | |
|---|---|---|---|---|---|
| Tumour size | ≥5 cm | 11 | 27 | 7 | NS |
| | <5 cm | 8 | 35 | 12 | |
| Portal invasion | with | 9 | 19 | 8 | NS |
| | without | 10 | 43 | 11 | |
| Intrahepatic metastasis | with | 2 | 18 | 3 | NS |
| | without | 17 | 44 | 16 | |
| Carcinoma differentiation | poor | 6 | 13 | 7 | NS |
| moderate or well | 13 | 49 | 12 | | |

DFS of the patients (Kapitanovic et al, 2000). On the other hand, such relationships have not been found with other neoplasms such as carcinoma of the esophagus (Freiss et al, 1999), stomach (Sanidas et al, 1993) and ovary (Simpson et al, 1995). In the prostate, a recent study demonstrated the frequent expression of c-erbB-3 in prostatic intraepithelial neoplasia as well as prostatic carcinoma, suggesting that c-erbB-3 plays a role in the carcinogenesis of this carcinoma (Haussler et al, 1999). Therefore, the role of c-erbB-3 may not be uniform for all neoplasms, and HCC displays c-erbB-3 expression as a typical characteristic.

c-erbB-4 protein expression in carcinoma nests was usually equal to or less than in noncancerous lesions. A similar tendency was obtained for other carcinomas such as those of the breast, colon, ovary, bronchus, and head and neck (Srinivasan et al, 1998), although the opposite tendency was obtained for thyroid carcinoma (Haugen et al, 1996), endometrial carcinoma (Srinivasan et al, 1999), astrocytoma (Srinivasan et al, 1998) and cholangiocellular carcinoma (Ito et al, in press). Among them, breast carcinoma has been comparatively well-studied for c-erbB-4 expression, which is stronger in more differentiated breast carcinoma cell lines than in the more aggressive ones (Plowman et al, 1993; Suo et al, 1998), in normal epithelia than in carcinomas (Srinivasan et al, 1998, 2000; Suo et al, 1998), and in cases with steroid receptors than in those without them (Riese et al, 1996). Furthermore, more recent studies demonstrated that c-erbB-4 expression is associated with a more differentiated histological phenotype (Kew et al, 2000; Srinivasan et al, 2000). On the other hand, our present study with HCC demonstrated that c-erbB-4 expression is not linked to any clinicopathological features including disease-free survival of the patients. Therefore, the clinical significance of c-erbB-4 in HCC is not clear and still needs to be elucidated. Some ligands of c-erbB-4 such as heparin binding epidermal growth factor-like growth factor (HB-EGF), batacellulin, epiregulin and neuregulins have been identified (Riese et al, 1996; Elenius et al, 1997; Komurasaki et al, 1997; Zhang et al, 1997). Investigations regarding the expression of these ligands, which have been partially done for endometrial carcinoma (Srinivasan et al, 1999), and regarding the status of signal transduction via c-erbB-4 when the ligands do or do not bind to this receptor and when heterodimerization occurs between c-erbB-4 and other type I receptor family, should help clarify the significance of c-erbB-4 in HCC.

In this study, we found cytoplasmic immunoreactivity especially of c-erbB-3 and c-erbB-4, although they are also transmembranous glycoprotein receptors. The significance of this finding is still unclear, but one possible explanation is the delayed turnover of these receptors in the cytoplasm. Baulida et al demonstrated that EGF-R is internalized and rapidly down- regulated subject to lysosomal degradation when it forms a complex with EGF, while such a ligand-induced mechanism could not be observed in other type I receptors (Baulida et al, 1996). Furthermore, of the type I family, EGF-R only has internalization codes within its cytoplasmic domain (Chang et al, 1993). These findings may, at least in part, explain the cytoplasmic localization of c-erbB-3 and c-erbB-4 in contrast to the dominant membranous localization of EGF-R. Alternatively, these proteins may accumulate in the cytoplasm as a latent form in larger quantity than EGF-R and c-erbB-2 before they become active at the cell membrane. This point needs to be further specified by future studies from different aspects. A recent study has demonstrated the nuclear localization of c-erbB-4 (Srinivasan et al, 1999, 2000; Kew et al, 2000), but we did not find this in our HCC series.

In the present study, we also demonstrated that EGF-R expression is directly linked to c-erbB-3 expression. This phenomenon was found in previous in vitro studies as well as a clinical study using surgical specimens for transitional cell carcinoma (Kim et al, 1994; Soltoff et al, 1994; Chow et al, 1997). Further studies are needed to find whether the relationship is due to heterodimerization of these receptors in this carcinoma.

In summary, the present study showed that, of the four kinds of type 1 family receptors, EGF-R and c-erbB-3 have roles in the progression of HCC. Further studies are needed to elucidate the meaning of the less frequent expression of c-erbB-2 and reduced expression of c-erbB-4 in HCC as compared to noncancerous lesions.

### REFERENCES

Bacus SS, Chin D, Yarden Y, Zemick CR and Stern DF (1996) Type 1 receptor tyrosine kinases are differentially phosphorylated in mammary carcinoma and differentially associated with steroid receptors. *Am J Pathol* **148**: 549–558

Baulida J, Kraus MH, Alimandi M, Di Fiore PP and Carpenter G (1996) Al Erb B receptors other than the epidermal growth factor receptor are endocytosis impaired. *J Biol Chem* **271**: 5251–5257

Beasley RP, Hwang L-Y, Lin C-C and Chien C-S (1981) Hepatocellular carcinoma and hepatitis B virus. *Lancet* **11**: 1129–1133

Berchunk A, Kamel A, Whitaker R, Kerns B, Olt G, Kinney R, Soper JT, Dodge R, Clarke-Pearson DL and Marks P (1990) Overexpression of HER-2/neu is associated with poor survival in advanced epithelial ovarian cancer. *Cancer Res* **50**: 4087–4091

Bobrow LG, Millis RR, Happerfield LC and Gullick WJ (1997) c-erbB-3 protein expression in ductal carcinoma in situ of the breast. *Eur J Cancer* **33**: 1846–1850

Bodey B, Bodey B Jr, Groger AM, Luck JV, Siegel SE, Taylor CR and Kaiser HE (1997) Clinical and prognostic significance of the expression of the c-erbB-2 and c-erbB-3 oncoproteins in primary and metastatic malignant melanomas and breast carcinomas. *Anticancer Res* **17**: 1319–1330

Borg A, Tandom AK, Sigurdsson H, Clark GM, Ferno M, Fuqua SA, Killander D and McGuire WL (1990) HER-2/neu amplification predicts poor survival in node-positive breast cancer. *Cancer Res* **50**: 4332–4337

Brunt EM and Swanson PE (1992) Immunoreactivity for c-erbB-2 oncopeptide in benign and malignant diseases of the liver. *Am J Clin Pathol* **97**(suppl 1): S53–61

Cantley LC, Auger KR, Carpenter C, Duckworth B, Graziani A, Kapeller R and Soltoff S (1991) Oncogenes and signal transduction. *Cell* **64**: 281–302

Chang C-P, Lazar CS, Walsh BJ, Komuro M, Collawn JF, Kuhn LA, Tainer JA, Trowbridge IS, Farguhar MG, Rosenfeld MG, Wiley HS and Gill GN (1993) Ligand-induced internalization of the epidermal growth factor receptor is mediated by multiple endocytic codes analogous to the tyrosine motif found in constitutively internalized receptors. *J Biol Chem* **268**: 19312–19320

 *© 2001 Cancer Research Campaign*

Chomczynski P and Sacchi N (1987) Single-step method of RNA isolation by acid guanidium thiocyanate-phenol-chloroform extraction. *Anal Biochem* **162**: 156–159

Chow N-H, Liu H-S, Yang H-B, Chan S-H and Su I-J (1997) Expression patterns of erbB receptor family in normal urothelium and transitional cell carcinoma. *Virchows Arch* **430**: 461–466

Collier JD, Guo K, Mathew J, May FEB, Bennett MK, Corbett IP, Bassendine MF and Burt AD (1992) c-erbB-2 oncogene expression in hepatocellular carcinoma and cholangiocarcinoma. *J Hepatol* **14**: 377–380

Dugan MC, Dergham ST, Kucway R, Siingh K, Biernat L, Du W, Vaitkevicius VK, Crissman JD and Sarkar FH (1997) HER-2/neu expression in pancreatic adenocarcinoma: relation to tumor differentiation and survival. *Pancreas* **14**: 229–236

Elenius K, Paul S, Allison G, Sun J and Klagsbrum M (1997) Activation or HER4 by heparin binding EGF-like growth factor stimulates chemotaxis but not proliferation. *EMBO J* **16**: 1268–1278

Freiss H, Fukuda A, Tang W-H, Tang W-H, Eichenberger A, Furlan N, Zimmermann A, Korc M and Buchler MW (1999) Concomitant analysis of the epidermal growth factor receptor family in esophageal cancer: overexpression of epidermal growth factor receptor mRNA but not of c-erbB-2 and c-erbB-3. *World J Surg* **23**: 1010–1018

Haugen DRF, Akslen LA, Varhaug JE and Lillehaug JR (1996) Expression of c-erbB-3 and c-erbB-4 proteins in papillary thyroid carcinomas. *Cancer Res* **56**: 1184–1188

Haussler O, Epstein JI, Amin MB, Heitz PU and Hailemariam S. (1999) Cell proliferation, apoptosis, oncogene, and tumor suppressor gene status in adenosis with comparison to benign prostatic hyperplasia, prostatic intraepithelial neoplasia, and cancer. Human Pathol **30**: 1077–1086

Ibrahim SO, Vasstrand EN, Liavaag PG, Johannessen AC and Lillehaug JR (1997) Expression of c-erbB proto-oncogene family members in squamous cell carcinoma of the head and neck. *Anticancer Res* **17**: 4539–4546

Ito Y, Matsuura N, Sakon M, Takeda T, Umeshita K, Nagano H, Nakamori S, Dono K, Tsujimoto M, Nakahara M, Nakao K and Monden M (1999) Both cell proliferation and apoptosis significantly predict shortened disease-free survival in hepatocellular carcinoma. *Br J Cancer* **81**: 747–751

Ito Y, Takeda T, Sasaki Y, Sakon M, Yamada T, Ishiguro S, Imaoka S, Tsujimoto M, Higashiyama S, Monden M and Matsuura N. Expression and clinical significance of the erbB family in intrahepatic cholangiocellular carcinoma. *Pathology Res Pract* in press

Kameda T, Yasui W, Yoshida K, Tsujino T, Nakayama H, Ito M, Ito H and Tahara E (1990) Expression of ERBB2 in human gastric carcinomas: relationship between p185ERBB2 expression and the gene amplification. *Cancer Res* **50**: 8002–8009

Kapitanovic S, Radosevic S, Slade N, Kapitanovic M, Andelinovic S, Ferencic Z, Tavassoli M, Sparenti S, Pavelic K and Sparenti R (2000) Expression of erbB-3 protein in colorectal adenocarcinoma: correlation with poor survival. *J Cancer Res Clin Oncol* **126**: 205–211

Kern JA, Schwartz DA, Nordberg JE, Weiner DB, Greene ML, Torney L and Robinson RA (1990) p185neu expression in human lung adenocarcinomas predicts shortened survival. *Cancer Res* **50**: 5184–5187

Kew TY, Bell JA, Pinder SE, Denley H, Srinivasan R, Gullick WJ, Nicholson RI, Blamey RW and Ellis IO (2000) c-erbB-4 protein expression in human breast cancer. *Br J Cancer* **82**: 1163–1170

Kim HH, Sierke SL and Koland JG (1994) Epidermal growth factor-dependent association of phosphatidylinositol 3-kinase with the erbB3 gene product. *J Biol Chem* **269**: 24747–24755

Kira S, Nakanishi T, Suemori S, Kitamoto M, Watanabe Y and Kajiyama G (1997) Expression of transforming growth factor alpha and epidermal growth factor receptor in human hepatocellular carcinoma. *Liver* **17**: 177–182

Komurasaki T, Toyoda H, Uchida D and Morimoto S (1997) Epiregulin binds to epidermal growh factor receptor and ErbB-4 and induces tyrosine phosphorylation of epidermal growth factor receptor, ErbB-2, ErbB-3 and ErbB-4 *Oncogene* **15**: 2841–2848

Kraus MH, Issing W, Miki T, Popescu NC and Aaronson SA (1989) Isolation and characterisation of c-erbB-3, a third member of the erbB/epidermal growth factor receptor family; evidence of over expression in a subset of human mammary tumours. *Proc Natl Acad Sci USA* **86**: 9193–9197

Lee CS and Pirdas A (1995) Epidermal growth factor receptor immunoreactivity in gallbladder and extrahepatic biliary tract tumours. *Path Res Pract* **191**: 1087–1091

Liver Cancer Study Group of Japan (1992) *The General Rules for the Clinical and Pathological Study of Primary Liver Cancer* (3rd Edition), Kanehara Press: Tokyo

Nakopoulou L, Stefanaki K, Filaktopoulos D and Giannopoulou I (1994) C-erb-B-2 oncoprotein and epidermal growth factor receptor in human hepatocellular carcinoma: An immunohistochemical study. *Histol Histopath* **9**: 677–682

Pinkas-Kramarski R, Soussan L, Waterman H, Levkowitz G, Alroy I, Klapper L, Lavi S, Sager R, Ratzkin BJ, Sela M and Yarden Y (1996) Diversification of Neu differentiation factor and epidermal growth factor signaling by combinatorial receptor interactions. *EMBO J* **15**: 2452–2467

Plowman GD, Culouscou J-M, Whitney GS, Green JM, Carlton GW, Foy L, Neubauer MG and Shoyab M (1993) Ligand-specific activation of HER4/p180, a fourth member of the epidermal growth factor receptor family. *Proc Natl Acad Sci USA* **90**: 1746–1750

Riese DJ, Bermingham Y, van Raaij TM, Buckely S, Plowman GD and Stern DF (1996) Betacellulin activates the epidermal growth factor receptor and erbB-4, and induces cellular response patterns distinct from those stimulated by epidermal growth factor or neuregulin *Oncogene* **12**: 345–353

Saito I, Miyamura T, Ohbayashi A, Harada H, Katayama T and Kikuchi S (1990) Hepatitis C virus infection is associated with the development of hepatocellular carcinoma. *Proc Nathl Acad Sci USA* **87**: 6547–6549

Sambrook J, Fritsch EF and Maniatis T (1989) Molecular cloning: a laboratory manual (Edn 2.) Cold Spring Harbor, NY: Cold Spring Harbor Laboratory Press

Sanidas EE, Filipe MI, Linehan J, Lemoine NR, Gullick WJ, Rajkumar T and Levison DA (1993) Expression of the c-erbB-3 gene product in gastric cancer. *Int J Cancer* **54**: 935–940

Sasaki K, Murakami T and Kawasaki M (1987) The cell cycle associated change of the Ki-67 reactive nuclear antigen expression. *J Cell Physiol* **133**: 579–584

Shintani S, Funayama T, Yoshihama Y, Alcalde R, Ootsuki K, Terakado N and Matsumura T (1995) Expression of c-erbB family gene products in adenoid cystic carcinoma of salivary glands: an immunohistochemical study. *Anticancer Res* **15**: 2623–2626

Simpson BJB, Phillips HA, Lessells AM, Langdon SP and Miller WR (1995) c-erbB growth-factor-receptor proteins in ovarian tumours. *Int J Cancer* (Pred. Oncol.) **64**: 202–206

Soltoff SP, Carraway KL, Prigent SA, Gullick WG and Cantley LC (1994) ErbB3 is involved in activation of phosphaidylinositol 3-kinase by epidermal growth factor. *Mol Cell Biol* **14**: 3550–3558

Srinivasan R, Poulsom R, Hurst HC and Gullick W (1998) Expression of the c-erbB-4/HER-4 protein and mRNA in normal human fetal and adult tissues and in a survey of the nine solid tumour types. *J Pathol* **185**: 236–245

Srinivasan R, Benton E, McCormick F, Thomas H and Gullick WJ (1999) Expression of the c-erbB-3/HER-3 and c-erbB-4/HER-4 growth factor receptors and their ligands, neuregulin-1 α, neuregulin-1 β, and betacellulin, in normal endometrium and endometrial cancer. *Clinical Cancer Res* **5**: 2877–2883

Srinivasan R, Gillett CE, Barnes DM and Gullick WJ (2000) Nuclear expression of the c-erbB-4/HER-4 growth factor receptor in invasive breast cancers. *Cancer Res* **60**: 1483–1487

Suo Z, Emilsen E, Tveit KM and Nesland JM (1998) Type 1 protein tyrosine kinases in benign and malignant breast lesions. *Histopathology* **33**: 514–521

Terada T, Ashida K, Endo K, Horie S, Maeta H, Matsunaga Y, Takashima K, Ohta T and Kitamura Y (1998) c-erbB-2 protein is expressed in hepatolithiasis and cholangiocarcinoma. *Histopathology* **33**: 325–331

Travis A, Pinder SE, Robertson JFR, Bell JA, Wencyk P, Gullick WJ, Nicholson RI, Poller DN, Blamey RW, Elston CW and Ellis IO (1996) C-erbB-3 in human breast carcinoma: expression and relation to prognosis and established prognostic indicators. *Br J Cancer* **74**: 229–233

Ullrich A and Schlessinger J (1990) Signal transduction by receptors with tyrosine kinase activity. *Cell* **61**: 203–212

Uwaifo AO and Bababunmi EA (1984) Liver carcinogenesis in tropical Africa. *IARC Sci Publ* **63**: 59–88

Yang J-L, Yu Y, Markovic B, Russell PJ and Crowe PJ (1997) Overexpression of c-erbB-2 mRNA and/or c-neu oncoprotein is a predictor for metastases from colorectal cancer. *Anticancer Res* **17**: 1023–1026

Zhang D, Sliwkowski MX, Mark M, Frantz G, Akita R, Sun Y, Hillan K, Crowley C, Brush J and Godowski PJ (1997) Neuregulin-3 (NRG3): a novel neural tissue-enriched protein that binds and activates ErbB4. *Proc Natl Acad Sci USA* **94**: 9562–9567

© 2001 Cancer Research Campaign 
